# Electrochemical activity of Samarium on starch-derived porous carbon: rechargeable Li- and Al-ion batteries

**DOI:** 10.1186/s40580-020-00221-y

**Published:** 2020-03-18

**Authors:** Kaiqiang Zhang, Tae Hyung Lee, Min-Ju Choi, Araz Rajabi-Abhari, Seokhoon Choi, Kyung Soon Choi, Rajender S. Varma, Ji-Won Choi, Ho Won Jang, Mohammadreza Shokouhimehr

**Affiliations:** 1 Department of Materials Science and Engineering, Research Institute of Advanced Materials, Seoul National University, Seoul, 08826 Republic of Korea; 2 Electronic Materials Center, Korea Institute of Science and Technology (KIST), Seoul, 136-791 Republic of Korea; 3 Program in Environmental Materials Science, Department of Forest Science, College of Agriculture and Life Sciences, Seoul National University, Seoul, Republic of Korea; 4 Advanced Nano-Surface Research Group, Korea Basic Science Institute, Daejeon, 34133 Republic of Korea; 5 Regional Center of Advanced Technologies and Materials, Department of Physical Chemistry, Faculty of Science, Palacky University, Š lechtitelů 27, 783 71 Olomouc, Czech Republic

**Keywords:** Starch, Samarium, Electrochemistry, Li-ion battery, Al-ion battery

## Abstract

Rechargeable metal-ion batteries are considered promising electric storage systems to meet the emerging demand from electric vehicles, electronics, and electric grids. Thus far, secondary Li-ion batteries (LIBs) have seen great advances in terms of both their energy and their power density. However, safety issues remain a challenge. Therefore, rechargeable Al-ion batteries (AIBs) with a highly reliable safety advantage and active electrochemical performances have gathered intensive attention. However, the common issue for these two metal-ion batteries is the lack of cathode materials. Many advanced electrode materials reported provide greatly enhanced electrochemical properties. However, their inherent disadvantages—such as complicated fabrication procedures, restricted manufacturing parameters, and the requirement of expensive instruments—limits their potential for further applications. In this work, we demonstrate the high electrochemical activity of the lanthanide element, Sm, towards storing charges when used in both LIBs and AIBs. Lanthanide elements are often overlooked; however, they generally have attractive electrochemical properties owing to their unpaired electrons. We employed starch as both a low-cost carbon source and as a three-dimensional support for Sm metal nanoparticles. The composite product is fabricated using a one-pot wet-chemical method, followed by a simultaneous carbonization process. As a result, highly improved electrochemical properties are obtained when it is used as a cathode material for both LIBs and AIBs when compared to bare starch-derived C. Our results may introduce a new avenue toward the design of high-performance electrode materials for LIBs and AIBs.

## Introduction

There have been great advancements in the development of batteries, highly efficient electric energy storage devices, with emerging application in electric vehicles, electronics, and electric grids [1 − 4]. Li-ion batteries (LIBs), among several others, have increased in both energy and power densities as a benefit of the scientific selection of elements and unique architectural designs of the electrodes [5–8]. Over time, other concerns regarding the development of LIBs, such as cost efficiency and practical availability, are gradually attracting attention. Thus, expended battery systems such as Na-, Mg, and Ca-ion batteries have been developed and display competitive electrochemical performances [9−14]. Al-ion batteries (AIBs) are one of these and have garnered attention in recent years because of their reliable safety, great cost efficiency, and excellent electrochemical properties [15 − 18]. Al metal can be directly inserted into the cell as an anode, which excludes the consideration of anode materials. This is quite a big difference between Al-ion battery systems and others. However, the lack of well-matched cathode materials inhibits the advance of these high-potential rechargeable AIBs. Recently, numerous nanostructured materials have been designed and synthesized for AIBs, displaying highly improved capacities, rate performance, and life-span [19 − 21]. To date, architectural carbon and sulfide materials still dominate the direction of research, although there have been some recent reports in the scientific literature. The exploration of promising cathode materials remains a challenge.

Carbon species is widely employed in battery chemistries serving as a conductive matrix with rich accessible active sites. Thus far, there have been reports on carbon-based electrodes for the structural design and functionalization process for both LIBs and AIBs [22, 23]. The exhausted researches sufficiently demonstrate the feasibility of carbon species used in battery chemistries. However, several common issues for the reported high-performance carbon electrodes are complex synthesis method, restricted manufacturing parameters, and expensive instrumental demand, which hinders the practical applications in terms of fabrication. Therefore, natural carbon sources without the need of special processing, are able to meet these demands. Polymers are promising candidates for the containment of rich carbon. After carbonization, a three-dimensional (3D) architectural and highly porous carbon matrix can be obtained. These factors are favorable to metal-ion batteries.

Starch is a green and natural polymer which can be facilely made by plant photosynthesis instead of laboratory fabrication with many toxic additives. In terms of raw material supply, it has an incomparable advantage over others. After carbonization, a 3D architecture can be formed that can facilitate the electrolyte penetration [24]. In addition, further engineering on the 3D carbon matrix is allowed.

Lanthanide elements exhibit quite interesting activity toward electrochemical reactions. Based on our previous discovery, the Gd element exhibits a highly enhanced capacity for Li ion storage when subjected to electrochemical systems [25]. Thus, the overlooked lanthanide elements with special 4*f* orbitals (lanthanide contraction) have stimulated further studies on their electrochemical properties.

Thus, in this work, we employed starch as the carbon source to synthesize a 3D carbon architecture and studied its electrochemical performances. As a result, the bare carbon exhibits a lower electrochemical capacity when used as a cathode material for LIBs and AIBs. Next, we reduced the Sm on the starch particles via a wet chemical method and followed it with a carbonization process. This one-pot prepared starch-derived carbon-supported Sm (SC/Sm) provides highly enhanced capacities as cathode materials in both LIBs and AIBs.

## Results and discussion

**Fig. 1:**
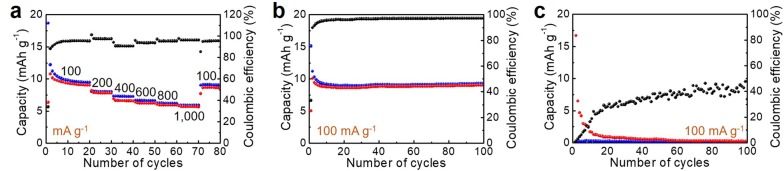
**a **Rate performance and **b** repeated charge/discharge properties of the SC as a cathode material of LIBs. **c** Repeated charge/discharge performance of SC as a cathode material of AIBs

The electrochemical performances of the SC were measured at different current densities (Fig. 1a). Lower capacity values were obtained for bare SC as the cathode material of LIBs, being 9.7, 8.2, 7.3, 6.7, 6.2, and 5.9 mAh g^−1^ at current densities of 100, 200, 400, 600, 800 and 1000 mA g^−1^, respectively. When the current density was enhanced up to 1000 mA g^−1^ from 100 mA g^−1^, 61% of the capacity was retained. Furthermore, the SC exhibited stable charge/discharge behaviors in the repeated charge/discharge measurement (Fig. 1b), with Coulombic efficiencies approaching 100%, although it had lower capacities. In addition, depressed capacities of the SC were shown when employed as the cathode materials of AIBs (Fig. 1c). After the initial phase, the capacities rapidly decayed to less than 3 mAh g^−1^. One main reason of the low capacity values when SC used as cathode of AIBs could be the narrow potential window. It is known that the redox active range of carbon in AIBs is around 2 V vs. AlCl_4_^−^/Al. These results strongly imply the suitability for subsequent demonstration of the electrochemical properties of Sm.

**Fig. 2:**
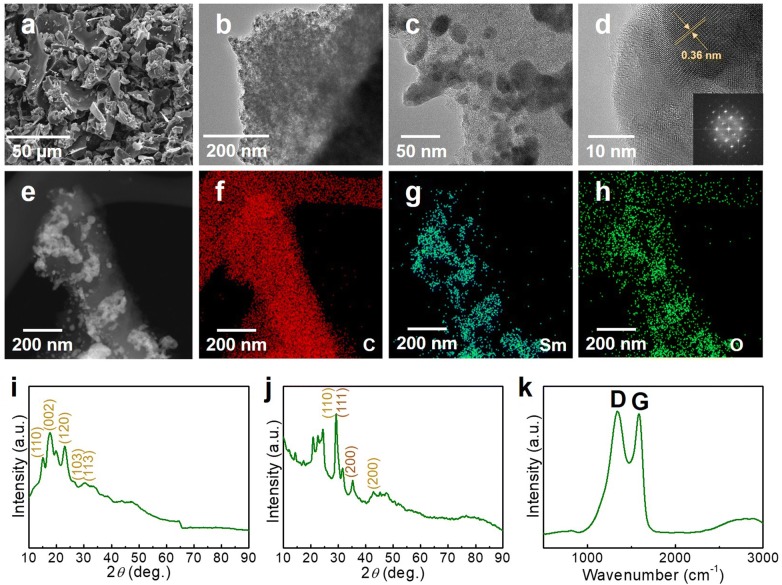
**a** SEM, **b**, **c** TEM, **d** HRTEM, **e** STEM, and **f**–**h** EDX mapping images of the SC/Sm. XRD patterns of **i** SC and **j** SC/Sm. **k** Raman spectrum of SC/Sm

Therefore, we engineered this SC by decorating Sm nanoparticles because they comprise rich unpaired electrons in the 4*f* orbital and could facilitate electrochemical reactions owing to their oxidation and reduction capabilities. The SC/Sm composite exhibits polyhedral morphologies with Sm particles immobilized on the carbon in the SEM image (Fig. 2a). As the magnification increases, a slightly porous 3D carbon matrix with a cotton-like morphology can be confirmed (Fig. 2b, c). The decorated Sm nanoparticles—approximately 30 nm in size—were trapped inside the porous carbon matrix, effectively inhibiting the agglomeration and loss of the Sm nanoparticles. In the HRTEM image (Fig. 2d), the presence of highly crystalline Sm nanoparticles with lattice interplanar spacing of approximately 0.36 nm were confirmed by the electron diffraction pattern (Fig. 2d, inset). The loaded Sm nanoparticles were further confirmed by EDX mapping (Fig. 2e−h). The highly porous carbon supports host the loaded Sm particles.

The crystallization of the Sm nanoparticles was confirmed by the XRD patterns. After loading Sm on starch, only carbon diffraction peaks were detected (Fig. 2i). After simultaneous carbonization and crystallization processes, the characteristic peaks (111) and (200) of Sm were seen together with the crystallized carbon (Fig. 2j), suggesting the existence of Sm crystals. The formation of carbon was further confirmed by the Raman spectrum (Fig. 2k). Here, the D band (A_1g_ mode) at 1344 cm^−1^ and G (E_2g_ mode) band at 1586 cm^−1^ suggest the consistency of the distorted and carbonized atoms. The co-existed D and G bands exhibit the similar phenomena with previously reported carbon-containing composite materials. Therefore, the natural starch is a promising carbon source in comparison with other synthesized materials.

**Fig. 3:**
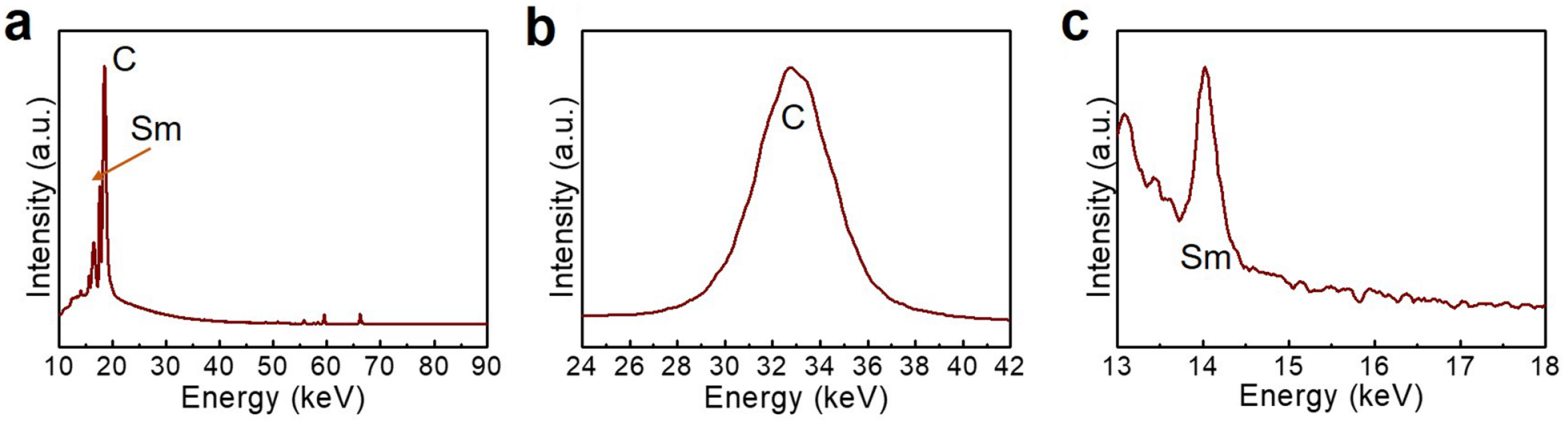
XRF spectra of **a** wide survey, **b** C, and **c** Sm of SC/Sm

The amount of Sm that was loaded on the SC was measured using the XRF technique. As a sequence, the introduced Sm is clearly shown by the intense peak in both the wide survey (Fig. 3a) and magnified spectrum (Fig. 3c), together with the C peak (Fig. 3b). This suggests the elemental configuration that we purposed. Furthermore, the elemental ratio of C and Sm is recorded in Table 1; it consisted of 52.6% C and 47.4% Sm.

**Table 1:** Sm loading amount in the SC/Sm measured by XRF

Component	Result (%)
C	*52.6*
Sm	*47.4*

**Fig. 4:**
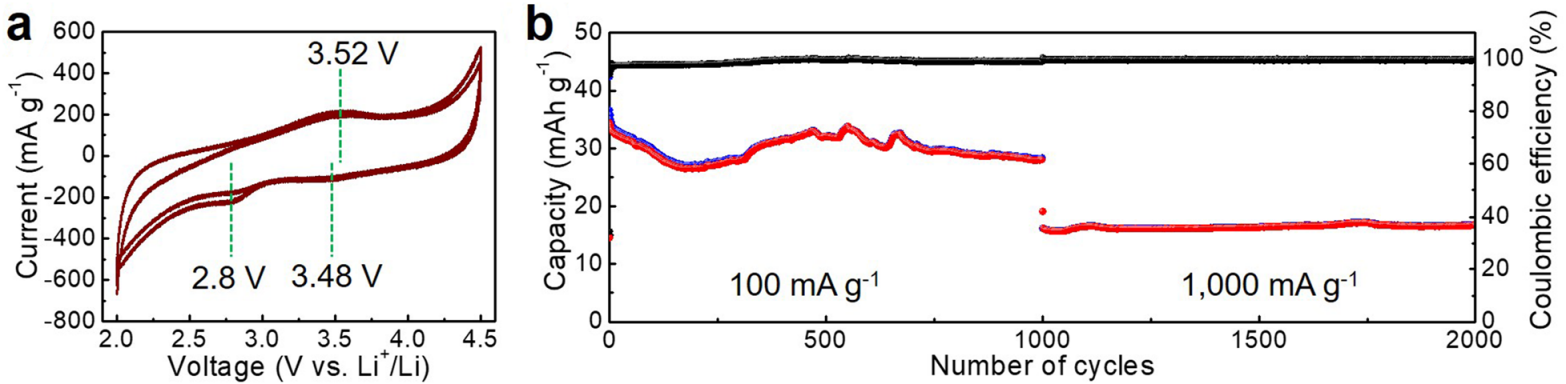
**a** CV curves and **b** repeated charge/discharge measurements for the SC/Sm as a cathode material of LIBs

The electrochemical activity of SC/Sm toward the Li ion storage is demonstrated by the CV curves (Fig. 4a), where a capacitance-type dominated charge storage process is illustrated by the rectangular CV curves in the potential window of 2.0–4.5 V vs. Li^+^/Li. The absence of noticeable redox peaks implies the limited redox rendering capability of introduced Sm particles. The resulted effects at the CV curves by loading Sm can be confirmed through the small redox peaks. The anodic peak at approximately 3.52 V vs. Li^+^/Li and the cathodic peaks at 2.8 and 3.48 V vs. Li^+^/Li are located on the CV curves, dedicating the redox activity of this composite toward the Li-ion storage. Furthermore, the repeated charge/discharge cycling measurement was conducted at two different current densities of 100 and 1000 mA g^−1^ (Fig. 4b). Highly enhanced capacity values (approximately 30 mAh g^−1^ at 100 mA g^−1^) in comparison with the bare SC (9.7 mAh g^−1^) were obtained, with Coulombic efficiencies approaching 100%. Furthermore, a retained capacity value of approximately 15 mAh g^−1^ was achieved when increasing the current density up to 1000 mA g^−1^. These results convincingly demonstrate that the introduced Sm can improve the Li-ion storage capability of the SC.

**Fig. 5:**
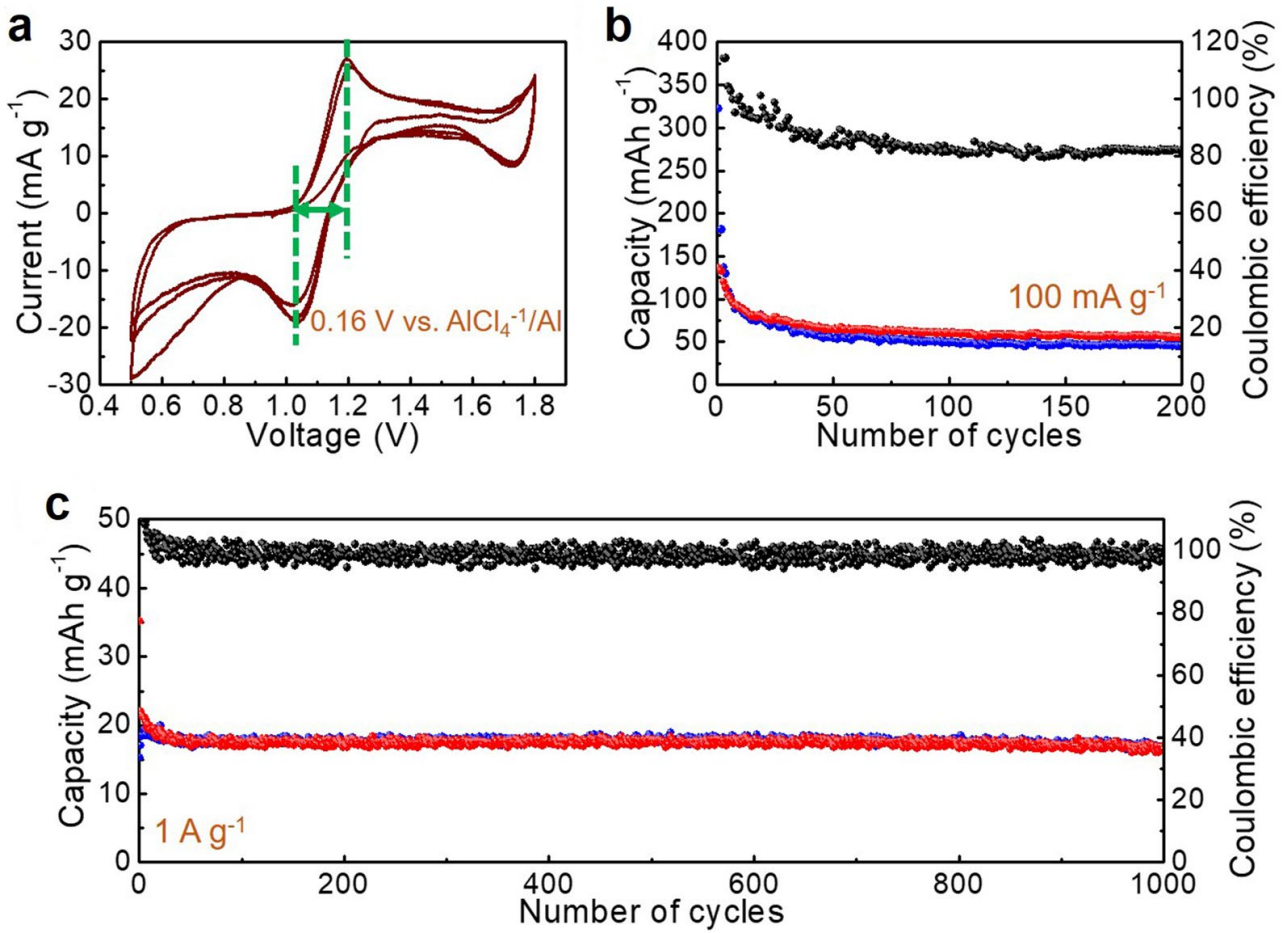
**a** CV curves and repeated charge/discharge measurements at **b** 100 mA g^−1^ and **c** 1,000 mA g^−1^ for the as-prepared SC/Sm as a cathode material of AIBs

Next, the composite SC/Sm used as a cathode material of AIBs was studied. As recorded in the CV curves (Fig. 5a), a pair of redox peaks implying the underlying charge storage are displayed at 1.2 (anodic) and 1.04 (cathodic) V vs. AlCl_4_^−^/Al, with a hysteresis voltage of 0.16 V vs. AlCl_4_^−^/Al. Afterward, repeated charge/discharge cycling measurements were conducted at current densities of 100 and 1000 mA g^−1^ (Fig. 5b, c), exhibiting steady charge/discharge capacity values. The Coulombic efficiency increases to approaching 100% when the current density is enhanced from 100 to 1000 mA g^−1^. Meanwhile, capacity values of approximately 50 mAh g^−1^ were obtained at 100 mA g^−1^ which are much higher than that of the bare SC. Furthermore, the capacity value of approximately 20 mAh g^−1^ is remained after enhancement of the specific current up to 1000 mA g^−1^.

**Fig. 6:**
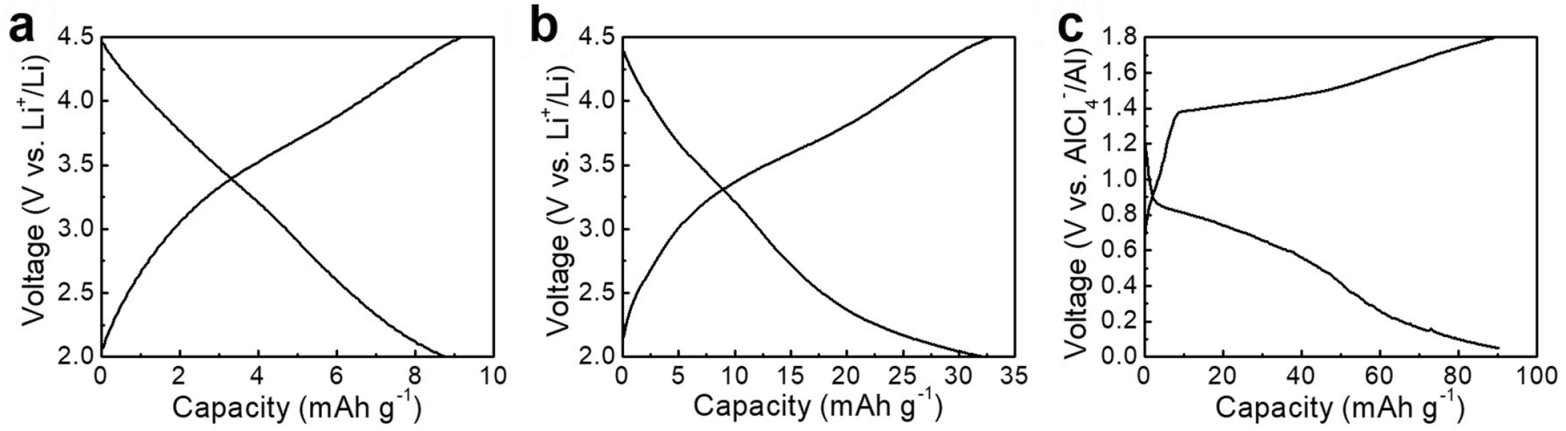
Voltage profiles of **a** bare C and **b** Sm/C when used for Li-ion storage. Voltage profiles of **c** Sm/C when used for Al-ion storage

The rendered redox activities of Sm loaded are further confirmed in the charge/discharge voltage profiles. The slope of discharge curve was changed after loading Sm when used as cathode for Li-ion storage (Fig. 6a, b). In addition, the noticeable voltage plateaus were located at 0.8 and 1.4 V vs. AlCl_4_^−^/Al, corresponding to the CV curves (Fig. 6c). The results evidence the rendering behavior of introduced Sm.

### As a anode material for Li-ion storage

**Fig. 7:**
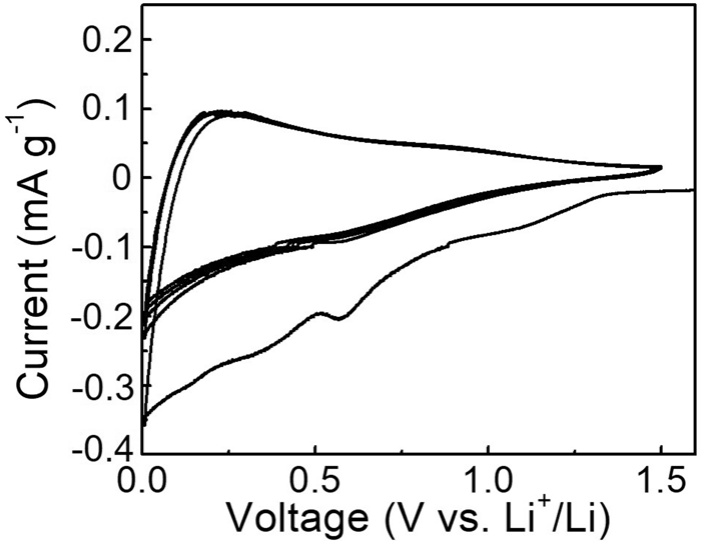
CV curves of Sm/C when used as anode for Li-ion storage

The ionic liquid electrolyte allowing stable plating and stripping of Al makes the feasibility of using Al foil as an anode directly. The lack of reliable safety when using Li foil as an anode leads the further study on anode materials of LIBs. In the demonstration of Sm/C as an anode material of LIBs, a capacitive-dominated charge storage process was confirmed, suggesting its limited activity (Fig. 7). At the first cycle of CV curves, the formation of solid electrolyte interface layer was conducted. Subsequently, tedious charge/discharge process is exhibited.

## Conclusions

In this work, we demonstrated the electrochemical activity of crystalline Sm nanoparticles toward the battery systems for both LIBs and AIBs. Starch was employed as a low-cost and eco-friendly carbon source to form a 3D and highly porous carbon matrix. The bare SC provides very limited capacity values when used as the cathode materials of both LIBs and AIBs. This unfavorable phenomenon can be modified by a simple decoration of the lanthanide element, Sm, nanoparticles. After which, the one-pot wet-chemical synthesized starch/Sm undergoes a simultaneous crystallization and carbonization process to form the final SC/Sm product. In comparison with the bare SC, highly improved electrochemical properties are obtained when it is modified into the cathode materials for both LIBs and AIBs. This work is expected to open a new avenue toward high-performance electrode materials for LIBs and AIBs.

## Experimental

### Material synthesis

1 g of starch and 0.1 g of cetyltrimethylammonium bromide (CTAB) were dissolved in a 30-mL aqueous solution with 10 mL of ethanol additive and then sonicated. Samarium (III) nitrate (0.01 mol) was dissolved in 10-mL de-ionized water. After sonication for 1 h, these two solutions were simply mixed and stirred constantly. Then 0.2 M of NaBH_4_ solution was added to the mixture [26–28]. After the reduction process, the suspended species was rinsed with copious water and dried in an oven at 60 °C for 8 h. Afterwards, the product obtained was carbonized at 900 °C for 5 h under Ar gas flow to form the 3D porous carbon-supported Sm nanoparticles. Furthermore, bare starch was also carbonized under the same conditions for an use as a reference material.

### Physical characterizations

The morphologies of the carbonized starch-supported Sm and bare starch were observed via field emission-scanning electron microscopy (FE-SEM, SUPRA 55VP). The 3D carbon matrix and loaded Sm nanoparticles were studied with a transmission electron microscope (TEM) coupled to an energy-dispersive X-ray spectrometer (EDX, JEOL JEM-F200). The crystalline Sm was confirmed by electron diffraction pattern and X-ray diffraction (XRD) with a Bruker D8 ADVANCE diffractometer. The composed elements were confirmed by energy dispersive EDX mapping. The features of the carbon matrix derived were measured based on Raman spectra obtained using a LabRAM HR Evolution and a Nicolet iS50. Furthermore, the amount of Sm that was loaded was measured via X-ray fluorescence spectroscopy (XRF, ZSX-PRIMUS).

### Electrode preparation

The mixed bare starch-derived carbon, carbon black (Super P Li), and poly(vinylidene difluoride) at a ratio of 7:2:1 were ground and dried for 8 h at 80 °C in a vacuum oven prior to further processing. The weight of the samples was measured before and after drying to ensure sufficient water evaporation. Afterward, the mixtures were re-dispersed into N-methyl-2-pyrrolidinone to produce a slurry. A working electrode was prepared by spreading the slurry on Cu, Al and Pt-deposited polymer foil current collectors with a loading amount of 1–3 mg cm^−2^, followed by drying in a vacuum oven at 60 °C for 8 h. The same methods were used for preparing the electrodes containing SC/Sm product by only changing the SC into SC/Sm.

### Electrochemical characterizations

#### As a cathode of LIBs

The electrochemical properties of both SC and SC/Sm were demonstrated in a two-electrode cell by inserting the cathodes comprising a SC or SC/Sm working electrode and sufficient Li metal foil anode in a LiPF_6_ electrolyte (1.0 M) with diethylene carbonate and ethylene carbonate (1:1, v/v) in a glovebox filled with Ar. Cyclic voltammetry (CV) curves were used to determine the electrochemical activity as conducted on an electrochemical workstation (WBCS3000, Wonatech, Korea). The potential range employed was 2.0 − 4.5 V vs. Li^+^/Li at a rate of 0.5 mV s^−1^. Galvanostatic charge/discharges were carried out in the same potential window of 2.0–4.5 V vs. Li^+^/Li with charge/discharge rates of 100, 200, 400, 600, 800, and 1000 mA g^−1^. The all-specific capacities and current densities were calculated based on the weight of the SC or SC/Sm.

#### As an anode of LIBs

Similar to the demonstration of Sm/C as a cathode of LIBs, the study of Sm/C as an anode of LIBs was performed. Cyclic voltammetry (CV) curves were used to determine the electrochemical activity as conducted on an electrochemical workstation (WBCS3000, Wonatech, Korea). The potential range employed was 0.05 − 1.5 V vs. Li^+^/Li at a rate of 0.5 mV s^−1^.

#### As a cathode of AIBs

The demonstration of SC and SC/Sm as cathode materials of AIBs was conducted in a pouch-type cell. The active material-containing electrode was inserted as a cathode. The cathode material and Al foil anode material were soaked in 1-Ethyl-3-methylimidazolium chloride/AlCl_3_ (EMIm[Cl]/AlCl_3_, 1/1.3 molar ratio) ionic liquid electrolyte. The assembly was conducted in a glovebox filled with Ar. The CV curves were scanned within the voltage range of 0.5–1.8 V vs. AlCl_4_^−^/Al, with a scan rate of 0.5 mV s^−1^. The galvanostatic charge/discharge was performed at current densities of 100 and 1000 mA g^−1^. The capacity values were calculated based on the weight of the SC or SC/Sm.

## Data Availability

The authors do not have other results to share as all data are shown in the present article.

## Abbreviations

LIBs: Li-ion batteries
AIBs: Al-ion batteries
3D: Three-dimensional
SC/Sm: Starch-derived carbon-supported Sm
HRTEM: High resolution transmission electron microscope
EDX: Energy-dispersive X-ray spectrometer
XRD: X-ray diffraction
XRF: X-ray fluorescence spectroscopy
CV: Cyclic voltammetry
CTAB: Cetyltrimethylammonium bromide
FE-SEM: Field emission-scanning electron microscopy
TEM: Transmission electron microscope
EMIm[Cl]/AlCl_3_: 1-Ethyl-3-methylimidazolium chloride/AlCl_3_
